# Transcription Factor MafB Suppresses Type I Interferon Production by CD14^+^ Monocytes in Patients With Chronic Hepatitis C

**DOI:** 10.3389/fmicb.2019.01814

**Published:** 2019-08-07

**Authors:** Tie-Mei Liu, Han Wang, Dong-Na Zhang, Guang-Ze Zhu

**Affiliations:** ^1^Department of Blood Transfusion and Department of Clinical Laboratory Medicine, China-Japan Union Hospital of Jilin University, Changchun, China; ^2^Department of Clinical Laboratory Medicine, The Affiliated Hospital to Changchun University of Chinese Medicine, Changchun, China

**Keywords:** hepatitis C virus, MafB, type I interferon, CD14^+^ monocytes, immunoregulation

## Abstract

Transcription factor MafB regulates differentiation and activity of monocytes/macrophage and is associated with the development of atherosclerosis and cancers. However, the role of MafB in modulation of CD14^+^ monocytes in chronic viral hepatitis was not fully elucidated. Thus, the aim of current study was to investigate the immunoregulatory function of MafB to type I interferon (IFN) secretion by CD14^+^ monocytes and its contribution to pathogenesis of chronic hepatitis C virus (HCV) infection. A total of 29 chronic hepatitis C patients and 21 healthy individuals were enrolled. Serum IFN-α1 and IFN-β was measured by ELISA, while MafB mRNA and protein expression were assessed by real-time PCR and Western blot. MafB siRNA or MafB expression plasmid was transfected into purified CD14^+^ monocytes to suppress or increase MafB expression. The function of MafB siRNA transfected CD14^+^ monocytes to HCV in cell culture (HCVcc)-infected Huh7.5 cells or CD4^+^ T cells was also investigated in direct and indirect contact co-culture system. Serum IFN-α1 and IFN-β was robustly reduced in chronic hepatitis C patients. By contrast, MafB was notably elevated in chronic hepatitis C patients and negatively correlated with serum IFN-α1. Overexpression of MafB reduced the IFN-α1 production by CD14^+^ monocytes from healthy individuals. However, MafB inhibition elevated IFN-α1 secretion by CD14^+^ monocytes and interferon regulatory factor 3 phosphorylation in chronic hepatitis C. MafB inhibition also promoted CD14^+^ monocytes-induced viral clearance in HCVcc-infected Huh7.5 cells by up-regulation of IFN-α1 and IFN-β without increasingly destroying hepatocytes, however, did not affect CD14^+^ monocytes-induced CD4^+^ T cells differentiation in chronic hepatitis C patients. The current data revealed that overexpression of MafB in chronic hepatitis C patients might suppress type I IFN production by CD14^+^ monocytes, leading to the viral persistence. MafB might be a potential therapeutic target for treatment of chronic hepatitis C.

## Introduction

Hepatitis C virus (HCV) chronically infects approximate 1.8 million persons all over the world, leading to millions of deaths due to the induction of end-stage liver diseases, such as decompensated liver cirrhosis, liver failure, and hepatocellular carcinoma (HCC; Negro, 2014; Lin et al., 2017; Yuan et al., 2017). Administration of oral direct-acting antiviral agents achieves high rate of sustained virological response, safety, and tolerability in chronic hepatitis C treatment (Falade-Nwulia et al., 2017). Nevertheless, there remains substantial HCV-associated disease burden due to the refractory and disparity in prevalence, transmission risk, and therapeutic response (Chhatwal et al., 2016). Thus, it is still important to not only increase the capacity of HCV screening and treatment but also further elucidate the pathogenesis of viral persistence.

Monocytes, macrophage, and dendritic cells are critical factors in innate immunity. Innate immune cells-secreting cytokines, such as interleukin (IL)-6, IL-12, and type I interferon (IFN-α1 and IFN-β), play important roles in shaping adaptive immunity by regulation of T cells differentiation during HCV infection (Saha and Szabo, 2014; Xu and Zhong, 2016; Yang et al., 2018). CD14^+^ monocytes are negatively correlated with memory CD4^+^ T cells and predict HCV-decline during therapy (Judge et al., 2016). Moreover, HCV infected monocytes show few cytopathic effects and continuously produce HCV as an amplification system (Revie and Salahuddin, 2014). By contrast, HCV infection activates monocytes-derived macrophages and induces IFN-α1/β expression *via* IFN regulatory factor (IRF) signaling pathway to suppress HCV replication in hepatocytes (Wang et al., 2015).

Transcription factor MafB is expressed by monocytes and macrophage and is involved in cellular differentiation and phagocytosis in macrophages (Tsuchiya et al., 2015). MafB enhances efferocytosis and promotes anti-inflammatory M2 polarization in macrophages (Kim, 2017; Sato et al., 2018). More importantly, MafB impairs the interaction of coactivator with IRF3 and downregulates IFN-α1/β production, leading to antiviral responses antagonization (Kim and Seed, 2010; Motohashi and Igarashi, 2010). Thus, we hypothesized that MafB-induced type I IFN reduction in CD14^+^ monocytes might also take part in the formation of viral persistence in patients with chronic hepatitis C. To test this possibility, we investigated the effect of MafB inhibition in purified CD14^+^ monocytes from chronic hepatitis C patients on IFN-α1/β production, viral replication, and CD4^+^ T cells differentiation *in vitro*.

## Patients, Materials, and Methods

### Subjects

The study protocol was approved by the Ethics Committee of the Affiliated Hospital to Changchun University of Chinese Medicine (No. 2016034) and China-Japan Union Hospital of Jilin University (No. JDSYLL-2017-001). Written informed consent was obtained from each enrolled subject. A total of 29 patients with chronic hepatitis C, and 21 healthy individuals with matched average age and sex ratio were enrolled in this study. All patients were positive for both anti-HCV and HCV RNA for at least 6 months and were hospitalized or followed-up in the Affiliated Hospital to Changchun University of Chinese Medicine or China-Japan Union Hospital of Jilin University between March 2017 and December 2017. No enrolled patients were co-infected with other hepatitis virus or human immunodeficiency virus (HIV) or afflicted with advanced liver diseases. Patients who received antiviral or immunomodulatory therapies before baseline sampling were excluded. The clinical characteristics of enrolled subjects were shown in Table 1.

**Table 1:** Clinical characteristics of enrolled subjects.

	Chronic hepatitis C	Healthy individuals
Cases (*n*)	29	21
Sex (male/female)	15/14	11/10
Age (years)	34 (18–61)^#^	35 (23–59)
HCV RNA (log_10_copies/ml)	6.54 ± 1.82	Not available
ALT (IU/L)	67 (17–388)^#^	27 (8–39)
HCV genotype (1b/2a/3)	12/11/6	Not available

# *Data were shown as median and range*.

### Virological Assessment

Anti-HCV antibody was measured using commercial enzyme immunoassay kits (Jinhao Biotech, Beijing, China). HCV RNA was quantified using commercial real-time PCR kits (PG Biotech, Shenzhen, Guangdong Province, China) with detection limitation of 2log_10_copies/ml. HCV genotyping was performed by line probe assay (Inno-Lipa, Innogenetics, Zwijndrecht, Belgium).

### CD14^+^ Monocytes and CD4^+^ T Cells Purification

Blood samples were collected from each enrolled subjects, and peripheral blood mononuclear cells (PBMCs) were isolated by density gradient centrifugation using Ficoll-Hypaque (Solarbio, Beijing, China). CD14^+^ monocytes were purified using human CD14 microbeads (Miltenyi Biotech, Bergisch Gladbach, Germany), while CD4^+^ T cells were purified using human CD4^+^ T cell isolation kit (Miltenyi) according to the instructions of manufacturer. The purity of enriched cells was more than 95% by flow cytometry determination.

### Transfection of MafB siRNA or MafB Expression Plasmid

siRNA specific for human MafB (sc-35839) and control siRNA (sc-37007) was purchased from Santa Cruz Biotechnology (Santa Cruz, CA, USA). About 0.5 μg of siRNA was transfected into CD14^+^ monocytes from chronic hepatitis C patients using Lipofectamine 2000 (Invitrogen Thermofisher, Carlsbad, CA, USA) according to the instructions of manufacturer. MafB opening reading frame was amplified from pSos-MafB plasmid (Stratagene, La Jolla, CA, USA) using Elongase *Taq* polymerase (Invitrogen Thermofisher) by polymerase chain reaction (PCR) and was ligated into pcDNA3.1/V5-His TOPO TA vector (Invitrogen Thermofisher). About 2 μg of pcDNA3.1/V5-His TOPO plasmid or 2 μg of pcDNA3.1/V5-His MafB plasmid was transfected into 2.5 × 10^6^ of CD14^+^ monocytes from healthy individuals using electroporation method for human monocytes by Nucleofector II (Amaxa Biosystems, Lonza, Basel, Switzerland). Cells and supernatants were harvested 48 h post-transfection for further experiments.

### Generation of Hepatitis C Virus Viral Stocks and Infection of Huh7.5 Cells

Infectious HCV in cell culture (HCVcc) was generated as described previously (Jiang et al., 2017). A 10^7^ copies of HCVcc were used to infect Huh7.5 cells. HCV RNA level in Huh7.5 cells was measured to confirm the infection.

### Cell Culture

CD14^+^ monocytes and CD4^+^ T cells were co-cultured in direct and indirect contact manners as previously described (Yang et al., 2017). In direct contact co-culture system, 10^5^ of CD14^+^ monocytes and 10^5^ of autologous CD4^+^ T cells were mixed directly in 24-well plates. In indirect contact co-culture system, 10^5^ of CD14^+^ monocytes were added into the upper chamber of Transwell plate (Corning, Corning, NY, USA), while 10^5^ of autologous CD4^+^ T cells were seeded into lower chamber. Thus, CD14^+^ monocytes and CD4^+^ T cells were separated by a 0.4 μm-pore membrane, which allowed the passage of soluble factors only. CD4^+^ T cells cultured alone were used as controls. In the last 6 h of the culture, phorbol-12-myristate-13-acetate (final dilution, 50 ng/ml), ionomycin (final dilution, 1 μg/ml), and Brefeldin A (final dilution, 10 μg/ml) was added for stimulation. In certain experiments, 10^5^ of CD14^+^ monocytes were co-cultured with 5 × 10^5^ of HCVcc infected Huh7.5 cells in both direct and indirect contact manners. Cells and supernatants were harvested 48 h post co-culture.

### Enzyme Linked Immunosorbent Assay

Cytokine expressions in serum and cultured supernatants were measured using commercial ELISA kits (Beyotime, Wuhan, Hubei Province, China) according to the instructions of manufacturer.

### Real-Time Polymerase Chain Reaction

Total RNA was extracted from cells using TriPure Isolation Reagent, RNA isolations (Roche LifeScience, Shanghai, China) according to the instructions of manufacturer. The first strand cDNA was synthesized using EvoScript Reverse Transcriptase (Roche LifeScience), and real-time PCR was performed using FastStart Essential DNA Green Master (Roche LifeScience). The relative gene expression was semi-quantified by 2^−ΔΔCT^ method LightCycler 96 instrument (Roche LifeScience). The sequences of primers were shown as follows: MafB forward: 5′-CAG AAG CAC CAC CTG GAG AAT-3′, MafB reverse: 5′-GAA CTC TGA CAG ACA GGT CCG-3′; β-actin forward: 5′-AGC GGG AAA TCG TGC GTG-3′, β-actin reverse: 5′-CAG GGT ACA TGG TGG TGC C-3′.

### Western Blot

Western blot was performed as previously described (Zhang et al., 2018). Briefly, cells were lysed on ice for 15 min in lysis buffer, and the supernatants were harvested by centrifugation at 12,000 × *g* for 10 min at 4°C. Total proteins were separated on SDS-PAGE using Mini-PROTEAN3 electrophoresis cell systems (Bio-Rad, Hercules, CA, USA) and were electroblotted onto PVDF membranes. The membranes were soaked in PBS containing 5% non-fat milk and 0.05% Tween 20 and were then incubated overnight in the presence of anti-MafB antibody (ab65953), anti-IRF3 antibody (ab25950), anti-IRF3 (phospho S386) antibody [EPR2346] (ab76493), or anti-β-actin antibody (ab8227). Membranes were washed five times after incubation, and HRP-conjugated goat anti-rabbit antibody IgG was added for an additional 2-h incubation. All antibodies were purchased from Abcam. Antigen-antibody complexes were visualized by enhanced chemiluminescence (Western Blotting Luminol Reagent, Santa Cruz, CA, USA).

### Target Cell Death Assay

Target HCVcc infected Huh7.5 cell death was determined by measuring lactate dehydrogenase (LDH) expression in the cultured supernatants using LDH Cytotoxicity Assay Kit (Beyotime) according to the instructions of manufacturer. LDH expression in HCVcc infected Huh7.5 cells was determined as low level control, while LDH expression in Triton X100-treated HCVcc infected Huh7.5 cells was determined as high level control. The percentage of target cell death was calculated as follows: (experiment value-low level control value)/(high level control value-low level control value) × 100%.

### Flow Cytometry

Cells were transferred into FACS tubes and were stained with anti-CD3-PE Cy7 (BD Bioscience, San Jose, CA, USA), anti-CD4-PerCP Cy5.5 (BD Bioscience) for a 30 min incubation in the dark at 4°C. Cells were fixed by adding 100 μl of Fixation & Permeabilization Medium A (Invitrogen Thermofisher) for a 15 min incubation in the dark at room temperature and were then resuspended in 100 μl of Fixation & Permeabilization Medium B (Invitrogen Thermofisher) containing anti-IFN-γ-APC (BD Bioscience), anti-IL-4-FITC (BD Bioscience), and anti-IL-17-PE (BD Bioscience) for another 20 min incubation. Isotype control antibodies were used to separate positive and negative cells in APC, FITC, and PE fluorescence channels. Samples were analyzed using FACS Aria II analyzer (BD Bioscience). Acquisitions were performed using CellQuest Pro Software (BD Bioscience), and analyses were performed using FlowJo Version 8.7.2 (Tree Star, Ashland, OR, USA).

### Statistical Analyses

Data were analyzed using SPSS Version 19.0 for Windows (Chicago, IL, USA). Shapiro-Wilk test was used for normal distribution assay. Variables following normal distribution were presented as mean ± standard deviation (SD). Statistical significance was determined by Student’s *t* test, paired *t* test, one-way ANOVA, or SNK-*q* test, and Pearson correlation analysis was used for correlation analysis. Variables following skewed distribution were presented as median [Q1, Q3]. Statistical significance was determined by Mann-Whitney test, Wilcoxon matched pairs test, Kruskal-Wallis test, or Dunn’s multiple comparison test, and Spearman correlation analysis was used for correlation analysis. All tests were two tails, and *p* less than 0.05 was considered to indicate significant differences.

## Results

### Serum IFN-α1 and IFN-β Was Downregulated in Chronic Hepatitis C Patients

**Figure 1:**
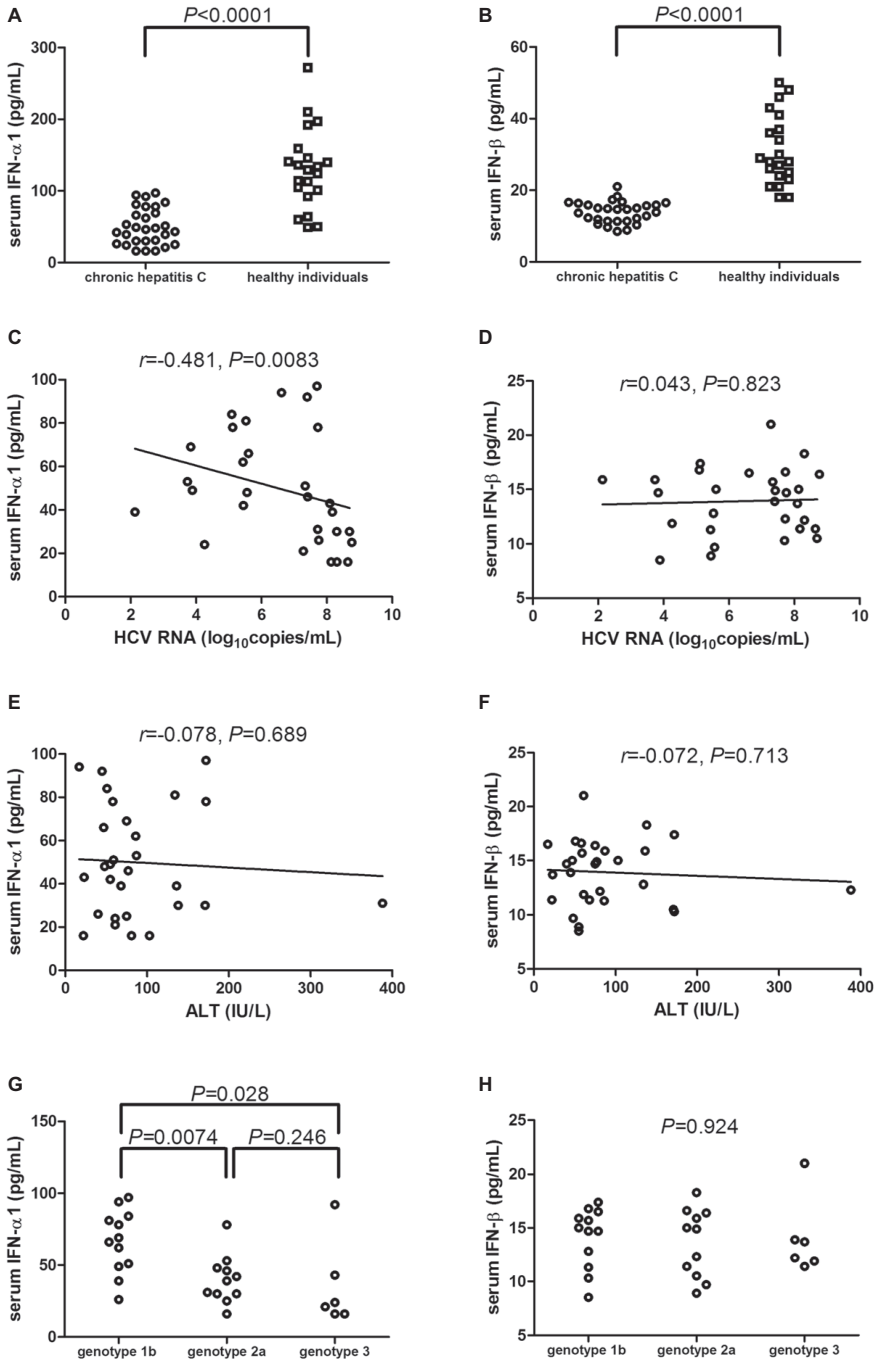
Serum IFN-α1 and IFN-β expression in chronic hepatitis C patients (*n* = 29) and healthy individuals (*n* = 21). IFN-α1 and IFN-β levels in the serum were measured by ELISA from all enrolled subjects. Comparison of **(A)** IFN-α1 and **(B)** IFN-β expression in the serum of chronic hepatitis C and healthy individuals. Significances were determined using Mann-Whitney test or Student’s *t* test. **(C)** Correlation between serum IFN-α1 and HCV RNA was determined using Spearman correlation analysis. **(D)** Correlation between serum IFN-β and HCV RNA was determined using Pearson correlation analysis. **(E)** Correlation between serum IFN-α1 and ALT was determined using Spearman correlation analysis. **(F)** Correlation between serum IFN-β and ALT was determined using Spearman correlation analysis. Comparison of **(G)** IFN-α1 and **(H)** IFN-β expression in the serum of different HCV genotypes. Significances were determined using Dunn’s multiple comparison test or one-way ANOVA. Individual level for each enrolled subject was shown.

IFN-α1 and IFN-β expression in the serum was measured by ELISA in all enrolled subjects. Serum IFN-α1 was significantly downregulated in chronic hepatitis C patients [46.86(28.42, 73.72) pg/ml] in comparison with healthy individuals [129.9(96.67, 152.1) pg/ml; Mann-Whitney test, *p* < 0.0001, Figure 1A]. Similarly, IFN-β level in the serum of chronic hepatitis C patients was also notably decreased when compared with healthy individuals (13.92 ± 3.01 vs. 31.10 ± 9.85 pg/ml; Student’s *t* test, *p* < 0.0001, Figure 1B). Serum IFN-α1 expression was negatively correlated with HCV RNA level (*r* = −0.481; Spearman correlation analysis, *p* = 0.0083, Figure 1C). However, there was no remarkable correlation between serum IFN-β and HCV RNA (*r* = 0.043; Pearson correlation analysis, *p* = 0.823, Figure 1D). Moreover, there were also no significant correlation between serum IFN-α1/β and ALT (Spearman correlation analysis, *p* > 0.05, Figures 1E,F). IFN-α1 expression in the serum of HCV genotype 2a patients [39.82(30.73, 48.04) pg/ml] and of HCV genotype 3 patients [22.33(16.50, 55.04) pg/ml] was significantly reduced than in HCV genotype 1b patients [67.33(49.96, 83.33) pg/ml; Dunn’s multiple comparison tests, *p* = 0.0074 and *p* = 0.028, respectively, Figure 1G]. However, there was no remarkable difference of serum IFN-β expression among chronic hepatitis C patients with different genotypes (one-way ANOVA, *p* = 0.924, Figure 1H).

### MafB mRNA and Protein Was Elevated in Chronic Hepatitis C Patients

**Figure 2:**
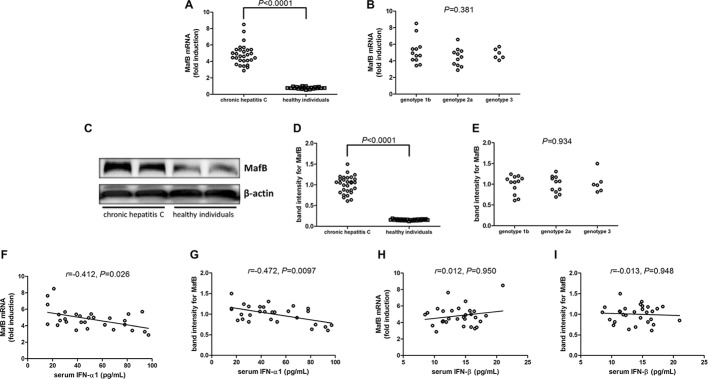
MafB mRNA and protein expression in chronic hepatitis C patients (*n* = 29) and healthy individuals (*n* = 21). MafB mRNA relative level was semi-quantified by real-time PCR. **(A)** Comparison of MafB mRNA relative level in PBMCs from chronic hepatitis C and healthy individuals. Significances were determined using Student’s *t* test. **(B)** Comparison of MafB mRNA relative level in PBMCs from different HCV genotypes. Significances were determined using one-way ANOVA. MafB protein expression was measured by Western blot. **(C)** Representative Western blot analysis was shown. **(D)** Comparison of MafB protein expression in PBMCs from chronic hepatitis C and healthy individuals. Significances were determined using Student’s *t* test. **(E)** Comparison of MafB protein expression in PBMCs from different HCV genotypes. Significances were determined using one-way ANOVA. **(F)** Correlation between MafB mRNA relative level and serum IFN-α1 was determined using Spearman correlation analysis. **(G)** Correlation between MafB protein expression and serum IFN-α1 was determined using Spearman correlation analysis. **(H)** Correlation between MafB mRNA relative level and serum IFN-β was determined using Spearman correlation analysis. **(I)** Correlation between MafB protein expression and serum IFN-β was determined using Spearman correlation analysis. Individual level for each enrolled subject was shown.

MafB mRNA relative level and protein expression in PBMCs were also measured in all enrolled subjects. MafB mRNA was approximate 5-flod elevation in PBMCs from chronic hepatitis C patients than those from healthy individuals (Student’s *t* test, *p* < 0.0001, Figure 2A). However, there was no significant difference of MafB mRNA relative level among chronic hepatitis C patients with different genotypes (one-way ANOVA, *p* = 0.381, Figure 2B). MafB protein expression was measured by Western blot, and representative Western blot analysis was shown in Figure 2C. The densities of the bands were semi-quantified, and intensity of MafB protein expression was calculated to the density of β-actin. MafB protein expression in PBMCs was also remarkably increased in chronic hepatitis C patients with approximately 10-fold elevation when compared with healthy individuals (Student’s *t* test, *p* < 0.0001, Figure 2D). Similar to MafB mRNA relative level, there was also no significant difference of MafB protein expression among chronic hepatitis C patients with different genotypes (one-way ANOVA, *p* = 0.934, Figure 2E). Interestingly, both MafB mRNA and protein level were negatively correlated with serum IFN-α1 expression in chronic hepatitis C patients (Spearman correlation analyses, *r* = −0.412, *p* = 0.026 and *r* = −0.472, *p* = 0.0097, respectively, Figures 2F,G). However, there were no remarkable correlations between MafB level (either mRNA or protein) and IFN-β expression in chronic hepatitis C patients (Pearson correlation analyses, *p* > 0.05, Figures 2H,I).

### MafB Inhibition Increased IFN-α1 Production by CD14^+^ Monocytes From Chronic Hepatitis C Patients

**Figure 3:**
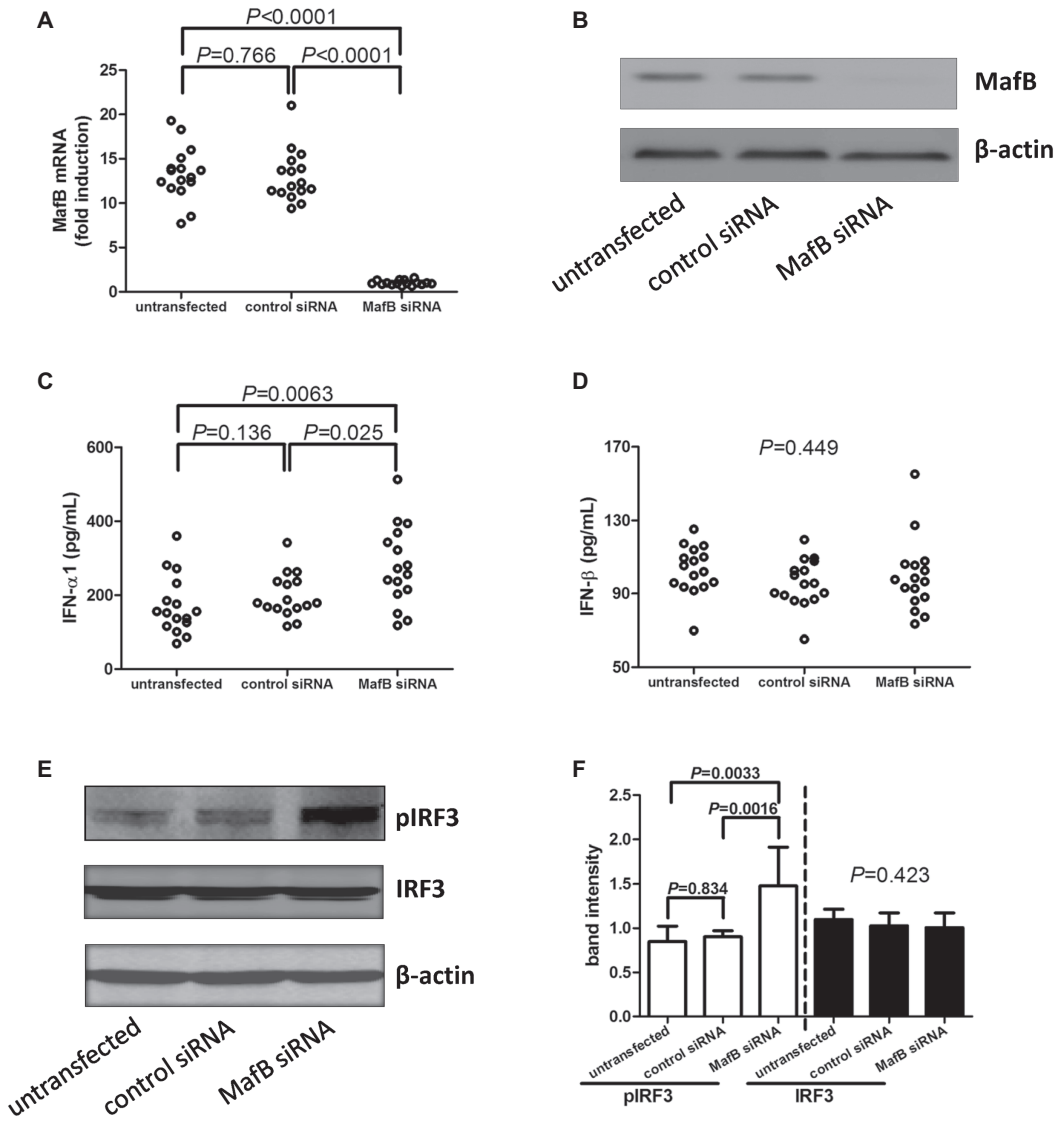
Type I IFN production and IRF3 phosphorylation by MafB inhibition in CD14^+^ monocytes from chronic hepatitis C patients (*n* = 16). CD14^+^ monocytes were transfected with control siRNA or MafB siRNA. Cells and supernatants were harvested 48 h post-transfection. **(A)** MafB mRNA relative level was semi-quantified by real-time PCR in untransfected, control siRNA transfected, and MafB siRNA transfected CD14^+^ monocytes. Significances were determined using SNK-*q* test. **(B)** MafB protein expression was measured by Western blot in untransfected, control siRNA transfected, and MafB siRNA transfected CD14^+^ monocytes. IFN-α1 and IFN-β levels in the cultured supernatants were measured by ELISA. Comparison of **(C)** IFN-α1 and **(D)** IFN-β expression in untransfected, control siRNA transfected, and MafB siRNA transfected CD14^+^ monocytes. Significances were determined using Dunn’s multiple comparison test or one-way ANOVA. **(E)** Phosphorylated IRF3 and total IRF3 was measured by Western blot in untransfected, control siRNA transfected, and MafB siRNA transfected CD14^+^ monocytes. **(F)** Comparison of IRF3 phosphorylation and total IRF3 in untransfected, control siRNA transfected, and MafB siRNA transfected CD14^+^ monocytes. Individual level for each enrolled subject was shown.

CD14^+^ monocytes were purified from 16 chronic hepatitis C patients, including eight genotype 1 patients, seven genotype 2a patients, and one genotype 3 patients. A 10^5^ of purified CD14^+^ monocytes were transfected with control siRNA or MafB siRNA, respectively. Cells and supernatants were harvested 48 h post-transfection. MafB siRNA transfected significantly reduced MafB expression in both mRNA (Figure 3A) and protein level (Figure 3B). Inhibition of MafB remarkably promoted IFN-α1 production [264.8(206.9, 362.9) pg/ml] by CD14^+^ monocytes in comparison with untransfected [154.4(118.6, 220.3) pg/ml; Dunn’s multiple comparison test, *p* = 0.0063, Figure 3C] and control siRNA transfected cells [179.4(164.1, 237.7) pg/ml; Dunn’s multiple comparison test, *p* = 0.024, Figure 3C]. However, MafB inhibition did not affect IFN-β secretion by CD14^+^ monocytes (one-way ANOVA, *p* = 0.449, Figure 3D). There was no statistical difference of pIRF3 expression in CD14^+^ monocytes between genotype 1b and genotype 2a patients (Mann-Whitney test, *p* > 0.05). Moreover, inhibition of MafB notably increased IRF3 phosphorylation in CD14^+^ monocytes (SNK-*q* test, *p* = 0.0016 and *p* = 0.0033 respectively, Figures 3E,F).

### MafB Inhibition Promoted CD14^+^ Monocytes-Induced Viral Clearance in HCVcc-Infected Huh7.5 Cells

**Figure 4:**
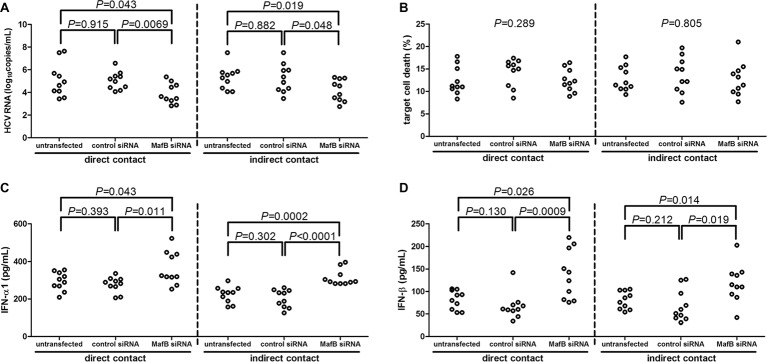
The influence of MafB inhibition to CD14^+^ monocytes from chronic hepatitis C patients (*n* = 10) on HCVcc infected Huh7.5 cells. CD14^+^ monocytes were transfected with control siRNA or MafB siRNA and were co-cultured with HCVcc infected Huh7.5 cells in direct and indirect contact manners for 48 h. Supernatants were harvested for further analysis. **(A)** HCV RNA was quantified by real-time PCR and was compared among groups. Significances were determined using SNK-*q* test. **(B)** Percentage of target cell death was determined by measuring LDH expression and was compared among groups. Significances were determined using one-way ANOVA. **(C)** IFN-α1 and **(D)** IFN-β were measured by ELISA and were compared among groups. Significances were determined using SNK-*q* test and Dunn’s multiple comparison test, respectively. Individual level for each enrolled subject was shown.

CD14^+^ monocytes were purified from 10 chronic hepatitis C patients, including six genotype 1 patients and four genotype 2a patients. CD14^+^ monocytes were transfected with MafB siRNA to suppress MafB expression. A 10^5^ of CD14^+^ monocytes were co-cultured in direct or indirect contact with 5 × 10^5^ of HCVcc infected Huh7.5 cells. Inhibition of MafB expression in CD14^+^ monocytes notably reduced HCV RNA in cultured supernatants in both direct (untransfected: 5.10 ± 1.49 log_10_copies/ml; control siRNA transfected: 5.04 ± 0.78log_10_copies/ml; MafB siRNA transfected: 3.90 ± 0.90 log_10_copies/ml; SNK-*q* test, *p* = 0.043 and *p* = 0.0069, respectively, Figure 4A) and indirect (untransfected: 5.31 ± 1.03 log_10_copies/ml; control siRNA transfected: 5.23 ± 1.26 log_10_copies/ml; MafB siRNA transfected: 4.17 ± 0.95 log_10_copies/ml; SNK-*q* test, *p* = 0.019 and *p* = 0.048, respectively, Figure 4A) contact co-culture system. However, there were no significant differences of target cell death in response to MafB siRNA transfected CD14^+^ monocytes in direct (one-way ANOVA, *p* = 0.289, Figure 4B) and indirect (one-way ANOVA, *p* = 0.805, Figure 4B) contact co-culture system. Furthermore, IFN-α1 and IFN-β expression was robustly elevated in cultured supernatants from direct and indirect contact systems in response to MafB inhibition (SNK-*q* test and Dunn’s multiple comparison test, all *p* < 0.05, Figures 4C,D). There were also no significant differences of cytokine production or target cell death between genotype 1b and genotype 2a patients (*p* > 0.05).

### MafB Inhibition Did Not Affect CD14^+^ Monocytes-Induced Differentiation of CD4^+^ T Cells in Chronic Hepatitis C Patients

**Figure 5:**
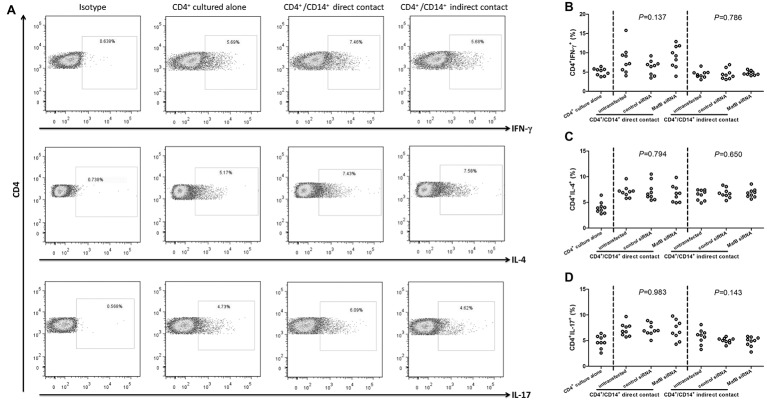
The influence of MafB inhibition to CD14^+^ monocytes from chronic hepatitis C patients (*n* = 9) on CD4^+^ T cells differentiation. CD14^+^ monocytes were transfected with control siRNA or MafB siRNA and were co-cultured with autologous CD4^+^ T cells in direct and indirect contact manners for 48 h. Cells were harvested for flow cytometry analysis. **(A)** Representative flow dots for CD4^+^IFN-γ^+^ (Th1 phenotype), CD4^+^IL-4^+^ (Th2 phenotype), and CD4^+^IL-17^+^ (Th17 phenotype) in MafB siRNA transfected CD14^+^ monocytes co-cultured with CD4^+^ T cells. Comparison of **(B)** CD4^+^IFN-γ^+^, **(C)** CD4^+^IL-4^+^, and **(D)** CD4^+^IL-17^+^ percentage among groups. Significances were determined using one-way ANOVA and SNK-*q* test. Individual level for each enrolled subject was shown.

It was well accepted that CD14^+^ monocytes were antigen presenting cells and could induce the differentiation of CD4^+^ T cells. Thus, the influence of MafB inhibition to CD14^+^ monocytes activity was investigated in direct and indirect contact co-culture system between CD14^+^ monocytes and CD4^+^ T cells. Representative flow dots for CD4^+^IFN-γ^+^ (Th1 phenotype), CD4^+^IL-4^+^ (Th2 phenotype), and CD4^+^IL-17^+^ (Th17 phenotype) were shown in Figure 5A. The percentages of Th1 and Th17 cells were significantly higher in direct contact co-culture system (SNK-*q* tests, *p* < 0.05, Figures 5B,D), but not in indirect contact co-culture system (SNK-*q* tests, *p* > 0.05, Figures 5B,D). By contrast, Th2 frequency was elevated in response to CD14^+^ monocytes induction in both systems (SNK-*q* tests, *p* < 0.05, Figure 5C). However, Inhibition of MafB did not affect CD4^+^ T cells differentiation induced by CD14^+^ monocytes in direct and indirect contact co-culture system (one-way ANOVA, *p* > 0.05, Figures 5B–D).

### Overexpression of MafB Suppressed IFN-α1 Production by CD14^+^ Monocytes From Healthy Individuals

**Figure 6:**
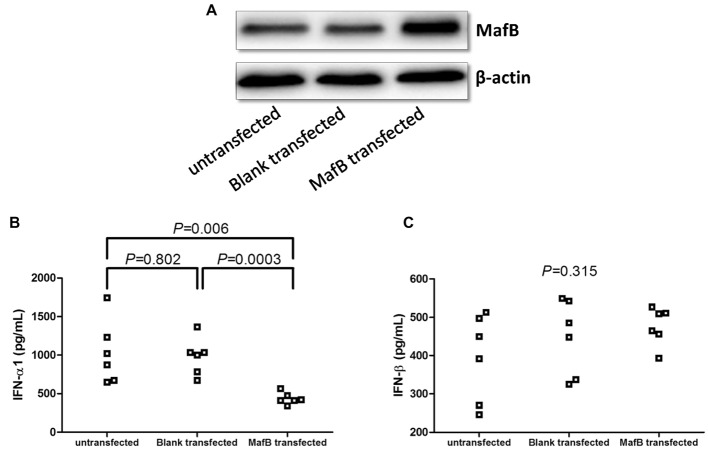
Type I IFN production by MafB expression plasmid transfection in CD14^+^ monocytes from chronic hepatitis C patients (*n* = 6). CD14^+^ monocytes were electroporated by blank plasmid or MafB expression plasmid. Cells and supernatants were harvested 48 h post stimulation. **(A)** MafB protein expression was measured by Western blot in untransfected, blank plasmid transfected, and MafB expression plasmid transfected CD14^+^ monocytes. Comparison of **(B)** IFN-α1 and **(C)** IFN-β expression in untransfected, blank plasmid transfected, and MafB expression plasmid transfected CD14^+^ monocytes. Significances were determined using one-way ANOVA and SNK-*q* test. Individual level for each enrolled subject was shown.

CD14^+^ monocytes, which were purified from six healthy individuals, were electroporated by blank plasmid or MafB expression plasmid. Transfection of MafB expression plasmid induced elevated MafB in CD14^+^ monocytes (Figure 6A). Overexpression of MafB reduced IFN-α1 production by CD14^+^ monocytes (436.9 ± 75.67 pg/ml) in comparison with untransfected (1,031 ± 411.9 pg/ml, SNK-*q* tests, *p* = 0.006, Figure 6B) and blank plasmid transfected cells (980.7 ± 239.9 pg/ml, SNK-*q* tests, *p* = 0.0003, Figure 6B), however, did not affect IFN-β secretion (One-Way ANOVA, *p* = 0.315, Figure 6C).

## Discussion

In the present study, elevated MafB negatively correlated with reduced serum IFN-α1 level in chronic hepatitis C patients. Inhibition of MafB in CD14^+^ monocytes purified from chronic hepatitis C patients induced elevated IFN-α1 secretion, which was accompanied by IRF3 phosphorylation. More importantly, inhibition of MafB promoted CD14^+^ monocytes-induced viral clearance in HCVcc-infected Huh7.5 cells probably *via* IFN-α1/β up-regulation without increasingly destroying infected hepatocytes. However, MafB inhibition did not affect CD14^+^ monocytes-induced CD4^+^ T cells differentiation *in vitro* in chronic hepatitis C patients. The current data suggested that MafB overexpression might suppress CD14^+^ monocytes-derived type I IFN production, which might be important in immune evasion and viral persistence during chronic HCV infection.

MafB was a lineage-specific transcription factor, which was highly and specifically expressed in CD14^+^ monocytes. Thus, MafB appeared to be one of the strong candidates for differentiation of monocytes/macrophages regulation due to the peculiar expression pattern and selective high-level expression in CD14^+^ cell context (Kelly et al., 2000; Montanari et al., 2005; Gemelli et al., 2006). MafB in CD14^+^ monocytes promoted tumorigenesis, cellular proliferation, and invasion in colorectal cancer (Yang et al., 2016), T cell acute lymphoblastic leukemia (Pajcini et al., 2017), nasopharyngeal carcinoma (Yang et al., 2015), and HCC (Yu et al., 2016). Human T-cell leukemia virus basic leucine-zipper factor abrogated DNA-binding activity and reduced the steady-state levels of MafB, leading to the disruption of transcriptional control of cellular genes and induction of adult T-cell leukemia (Ohshima et al., 2010; Reinke et al., 2010). Dysregulation of MafB was found in Zika and Dengue virus infection (Moni and Lio, 2017). To the best of our knowledge, the current results provided the first evidence of MafB expression in chronic HCV infection. We found that both MafB mRNA relative level and protein expression were robustly elevated and negatively correlated with reduced type I IFN production in the peripheral bloods of chronic hepatitis C patients, indicating that MafB might contribute to persistent HCV infection due to the important antiviral activity of IFN-α1/β.

Previous studies indicated that HCV infection selectively impaired type I IFN signaling, but not type II (IFN-γ) or type III (IFN-λ), which influenced antiviral responses in primary human hepatocytes (Chandra et al., 2014; Friborg et al., 2015). Type I IFN signaling was also a key driver for inflammation and immunosuppression in chronic infections and cancers (Snell et al., 2017). We also showed decreased expressions of IFN-α1 and IFN-β in the serum in chronic hepatitis C patients, and IFN-α1 was negatively correlated with HCV replication. This was consistent with the previous findings (Chandra et al., 2014). Type I IFNs were produced by almost all nucleated cells in response to viral infection (Motohashi and Igarashi, 2010). Reduced serum IFN-α1 in genotype 2a and genotype 3 but MafB levels in PBMCs are same as that of genotype 1b, although we found the negative correlation between MafB and IFN-α1. On the one hand, it might be partly due to the relative small size of the population, and large-scale number of chronic hepatitis C patients should be enrolled to confirm the current results. On the other hand, MafB was also shown to modulate the efficiency of IFN secretion by setting threshold for IRF3-dependent transcription (Kim and Seed, 2010; Motohashi and Igarashi, 2010). We did not found statistical difference of IRF3 phosphorylation between genotype 1b and genotype 2a patients. Thus, there might be other modulatory factors, which contributed to the differential expression of IFN-α1 expression among different genotypes. Due to the selective expression of MafB in CD14^+^ monocytes, we then further investigated the regulatory activity of MafB to CD14^+^ monocytes in chronic HCV infection and in healthy individuals. MafB siRNA transfection almost diminished mRNA and protein expression of MafB in purified CD14^+^ monocytes, indicating successful inhibition of MafB in CD14^+^ monocytes. Importantly, MafB inhibition directly promoted IFN-α1 production, but not IFN-β, by CD14^+^ monocytes from chronic hepatitis C patients, which was accompanied by IRF3 phosphorylation. In contrast, overexpression of MafB suppressed IFN-α1 production, but not IFN-β, by CD14^+^ monocytes from healthy individuals. The current results confirmed the previous finding that MafB was a negative regulator of type I IFNs and interfered with IRF3 activity (Kim and Seed, 2010). However, Kim and Seed showed that MafB mainly inhibited IFN-β activation, but not IFN-α1, which triggered by other IFN-β inducers, such as Newcastle disease virus infection in 293ETN cells (Kim and Seed, 2010). This might be partly due to the different infected cell types and varied infection states.

Although MafB deficiency directly facilitated type I IFN production and signaling, the role of MafB regulation to CD14^+^ monocytes function in chronic HCV infection was still unknown. HCV/HIV coinfection was associated with a type I IFN CD14^+^ monocytes activation profile and correlated with cognitive impairment (Rempel et al., 2013). Monocyte-derived macrophages mediated inhibition of HCV replication through secretion of type I IFN production *in vitro* (Wang et al., 2015). We set up direct and indirect contact co-culture system to distinguish cytolytic and non-cytolytic activity of CD14^+^ monocytes to HCVcc-infected Huh7.5 cells. MafB inhibition did not increase target cell death in both systems, indicating that MafB was not contribute to the cytolytic function of CD14^+^ monocytes. However, MafB inhibition in CD14^+^ monocytes exhibited increased activity for HCV suppression probably *via* elevation of IFN-α1 and IFN-β production, which presented as non-cytolytic activity. Thus, the current data suggested that MafB mainly suppressed type I IFN production by CD14^+^ monocytes in chronic HCV infection, and this impaired type I IFN secretion might contribute to persistent HCV infection. Furthermore, CD14^+^ monocytes also induced CD4^+^ T cells differentiation, which required direct cell-to-cell contact (Yang et al., 2017). This was consistent with the present findings, which showed that indirect cell contact between CD14^+^ monocytes and CD4^+^ T cells did not promote CD4^+^ T cells differentiation. By contrast, direct contact between CD14^+^ monocytes and CD4^+^ T cells elevated Th1, Th2, and Th17-phenotype differentiation. However, MafB inhibition did not affect this process, indicating MafB might dispensable for the immunoregulatory activity of CD14^+^ monocytes in chronic HCV infection.

There were several limitations of the current study. On the one hand, we only enrolled seven healthy individuals in MafB overexpression in CD14^+^ monocytes, because approximate 50 ml of peripheral bloods were needed for PBMCs isolation and CD14^+^ monocytes purification. On the other hand, most enrolled chronic hepatitis C patients did not followed-up in response to antiviral therapy. Thus, large-cohort studies with sufficient curative and followed-up observation periods are needed for further elucidation of MafB regulation to CD14^+^ monocytes during HCV infection.

In conclusion, HCV infection might lead to MafB overexpression, which suppressed type I IFN secretion by CD14^+^ monocytes. MafB-induced impaired CD14^+^ monocytes activity failed to inhibit HCV replication but was dispensable for the immunoregulation to CD4^+^ T cells, leading to the viral persistence. MafB might be a potential therapeutic target for treatment of chronic hepatitis C.

## Data Availability

The raw data supporting the conclusions of this manuscript will be made available by the authors, without undue reservation, to any qualified researcher.

## Ethics Statement

The studies involving human participants were reviewed and approved by the ethics committee of the Affiliated Hospital of Changchun University of Chinese Medicine and the ethics committee of China-Japan Union Hospital of Jilin University. The patients/participants provided their written informed consent to participate in this study.

## Author Contributions

G-ZZ designed and supervised the study. T-ML, HW, and D-NZ performed the experiments. T-ML and G-ZZ enrolled the patients. T-ML, HW, D-NZ, and G-ZZ analyzed the data. T-ML, D-NZ, and G-ZZ prepared the manuscript.

### Conflict of Interest Statement

The authors declare that the research was conducted in the absence of any commercial or financial relationships that could be construed as a potential conflict of interest.
